# Patient-reported outcome measures in the care of in-centre hemodialysis patients

**DOI:** 10.1186/s41687-021-00365-3

**Published:** 2021-10-12

**Authors:** Sara N. Davison, Scott Klarenbach, Braden Manns, Kara Schnick-Makaroff, Robert Buzinski, Bonnie Corradetti, Hilary Short, Jeffrey A. Johnson

**Affiliations:** 1 Division of Nephrology and Immunology, Department of Medicine, University of Alberta, 11-113L Clinical Sciences Building, Edmonton, AB T6G 2G3 Canada; 2 Department of Medicine and Department of Community Health Sciences, University of Calgary, O’Brien Institute of Public Health, 3280 Hospital Drive NW, Calgary, AB T2N 4Z6 Canada; 3 Faculty of Nursing, University of Alberta, 5-295 Edmonton Clinic Health Academy, Edmonton, AB T6G 1C9 Canada; 4 Patient Partner, Medicine Hat, Alberta, Canada; 5 Patient Partner, Calgary, AB Canada; 6 Alberta PROMs and EQ-5D Research and Support Unity, School of Public Health, University of Alberta, Shing Centre for Health Research Innovation, 2-040 Li Ka, Edmonton, AB T6G 2E1 Canada

**Keywords:** Patient reported outcome measures (PROMs), Hemodialysis, Edmonton Symptom Assessment System - Renal (ESAS-r:Renal), Health related qulity of life (HRQL)

## Abstract

Kidney failure requiring dialysis is associated with high symptom burden and low health-related quality of life (HRQL). Patient-reported outcome measures (PROMs) are standardized instruments that capture patients’ symptom burden, level of functioning, and HRQL. The routine use of PROMs can be used to monitor aspects of patients’ health that may otherwise be overlooked, inform care planning, and facilitate the introduction of treatments. Incorporating PROMs into clinical practice is an appropriate strategy to engage patients and enhance their role in decisions regarding their care and outcomes. However, the implementation of PROMs measurement and associated interventions can be challenging given the nature of clinical practice in busy hemodialysis units, the variations in organization and clinical workflow across units, as well as regional programs. Implementing PROMs and linking these with actionable treatment aids to alleviate bothersome symptoms and improve patients’ wellbeing is key to improving patients’ health. Other considerations in implementing PROMs within a hemodialysis setting include integration into electronic medical records, purchase and configuration of electronic tools (i.e., tablets), storage and disinfection of such tools, and ongoing IT resources. It is important to train clinicians on the practical elements of using PROMs, however there is also a need to engage clinicians to use PROMs on an ongoing basis. This article describes how PROMs have been implemented at in-centre hemodialysis units in Alberta, Canada, addressing each of these elements.

## Background

Patients with kidney failure requiring dialysis experience a high symptom burden and severity and low health-related quality of life (HRQL) [1]. However, their symptoms are often under-recognized and under-treated by their health care providers [2, 3]. A standardized and patient-centered process of symptom screening may improve symptom detection and treatment [2]. Patient-reported outcome measures (PROMs) are standardized instruments that capture patients’ reports of symptoms and impact of disease on functioning and/or HRQL [4]. Routine administration of PROMs can be used to evaluate and monitor aspects of patients’ health that may otherwise be overlooked, inform care planning, and facilitate the introduction of treatments [5, 6].

Historically, Alberta Health Services (AHS) has delivered kidney care through two programs covering the northern and southern regions of the province. In 2010, Alberta Kidney Care – North (AKC-N) introduced PROMs to in-centre hemodialysis units with the initiation of a supportive care program. This program was intended to identify patients with high palliative care needs who may benefit from additional supportive or palliative care services [7]. This involved routine screening of various indicators, including the modified Edmonton Symptom Assessment System (mESAS) [8, 9]. Patients completed the mESAS monthly; results were recorded in the electronic medical record (EMR), but not reviewed with the patient, nor tied to any specific guidance to inform clinical management.

Alberta Kidney Care – South (AKC-S) had not implemented PROMs until recently, when AKC-N partnered with AKC-S and the Ontario Renal Network to design, pilot, and rigorously evaluate PROMs interventions for in-centre hemodialysis care. These interventions were designed based on successes from PROMs studies in oncologic settings[10, 11] intending to enhance communication between patients and clinicians, maximize patient participation in decision-making on reducing symptoms, improve HRQL, and facilitate self-management [12]. From 2018—2020, a multi-centre cluster randomized controlled trial to evaluate the routine reporting of patient report outcomes in hemodialysis care, known as the EMPATHY trial, was implemented in in-centre hemodialysis units across Alberta and in select units in Ontario [13]. In part, the EMPATHY trial aimed to provide a deeper understanding of how to optimize the implementation and use of PROMs in hemodialysis [14, 15]. In this article, we aim to describe how PROMs are implemented in in-centre hemodialysis units in Alberta, Canada.

### Target population

PROMs are administered to adult patients undergoing chronic hemodialysis who are willing and able to complete the questionnaires. Patients with cognitive impairment, undergoing acute dialysis, or transiently dialyzing are not given PROMs. In some locations, PROMs were translated into the top three languages represented in the Alberta renal programs: Simplified Chinese, Punjabi, and Vietnamese. Patients with other language barriers either have a family member help, or the health system interpretive services are used. For patients with eyesight issues, their family members or nurses provide assistance. Most often, PROMs can be completed within 5 min but can vary depending on additional support required.

### PROMs selection

PROMs can be generic or disease-specific, and each has several advantages and disadvantages [16]. Generic measures are used to compare outcomes across different populations and interventions, particularly for cost-effectiveness studies and health system decision-makers. Disease-specific measures assess the health status of a specific patient population and may be more sensitive to detect important changes. A common recommendation in clinical research is to combine a generic and a disease-specific PROM for use in a particular patient population, especially when PROMs are used to monitor health over time. The presumed benefit of this combination is to broaden the scope of measurement for decision making at different levels (e.g., micro-level for patient management versus meso- or macro-levels). However, evidence to support this recommendation is limited. There is usually some overlap between generic and disease-specific measures, and the need for combining depends on the target patient population and the constructs assessed by each measure. It was important to explore whether one single PROM is sufficient to improve patient-provider communication, processes of care, and ultimately patient outcomes, before recommending the use of a combination of generic and disease-specific measures. The following measures were selected for evaluation through the EMPATHY trial (Table 1) [13]:

- Edmonton Symptom Assessment System Revised: Renal (ESAS-r: Renal) (for AKC-N)
- Integrated Palliative care Outcome Scale – Renal (IPOS-Renal) (for AKC-S)
- EQ-5D-5L

**Table 1:**
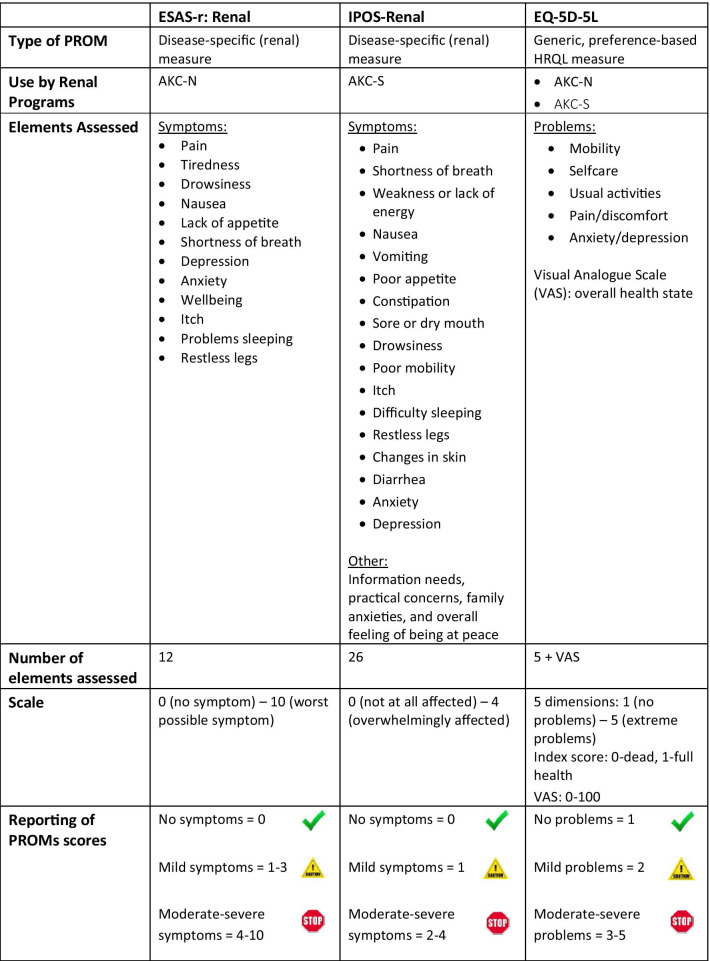
Comparison of PROMs

### Disease-specific PROMs

The Edmonton Symptom Assessment System (ESAS) is a clinically validated and reliable symptom measurement tool, extensively used in chronic diseases including cancer, heart disease, and kidney disease [17]. It was developed by the Regional Palliative Care Program, Capital Health in Edmonton, Alberta. The ESAS-r: Renal was modified from the original ESAS and validated for use with chronic kidney disease (CKD) patients in Canada to assess symptom burden [9]. While data from the ESAS-r: Renal had been previously collected in the AKC-N and adopted by other dialysis programs in Canada, use has not included systematic reporting of results to clinicians. The ESAS-r: Renal assesses 12 symptoms that bothered patients over the “past week” on a scale of 0 (no symptom) to 10 (worst possible symptom): pain, tiredness, drowsiness, nausea, lack of appetite, shortness of breath, depression, anxiety, wellbeing, itch, problems sleeping, and restless legs. The ESAS-r: Renal was implemented only within AKC-N.

The Palliative care Outcome Scale (POS) was developed in 1999[18] for use with patients with advanced disease, and to improve measurement of important outcomes in palliative care. The Integrated Palliative care Outcome Scale (IPOS) integrates questions from three different POS measures incorporating symptoms, information needs, practical concerns, family anxieties, and overall feeling of being at peace. The IPOS-Renal contains all the elements of the IPOS, and the most common symptoms renal patients experience: pain, shortness of breath, weakness or lack of energy, nausea, vomiting, poor appetite, constipation, sore or dry mouth, drowsiness, poor mobility, itch, difficulty sleeping, restless legs, changes in skin, diarrhea, anxiety, and depression. Each symptom is assessed based on how it affected patients “over the past week”. There are 26 items on a scale of 0 (not at all affected) to 4 (overwhelmingly affected). The IPOS-Renal has demonstrated validity and reliability and has been studied in dialysis and non-dialysis CKD patients in Australia [19]. The IPOS-Renal was implemented only within AKC-S.

### Generic PROM: EQ-5D-5L

Both renal programs in AHS implemented the EQ-5D-5L. The EQ-5D-5L is a generic preference-based measure of HRQL and has been selected as the PROM of choice in many clinical areas and settings. The EQ-5D-5L includes a health status classification system with five dimensions (mobility, self-care, usual activities, pain/discomfort, anxiety/depression), each with five levels of problems (1 = none, 2 = mild, 3 = moderate, 4 = severe, 5 = extreme), describing 3125 distinct health states [20]. An index score for each health state can be calculated using population preferences [21], which can then be used to estimate quality-adjusted life years in economic evaluations of health interventions or innovations [22]. Empirical evidence on the clinical application of the EQ-5D-5L with regards to individual patient care is limited, but a recent review of PROMs in CKD recommended the EQ-5D-5L for use in this patient population [23].

### PROMs collection

The presence and severity of symptoms can change considerably over a short period of time in hemodialysis patients, emphasizing the need for regular surveillance. Within the EMPATHY Trial protocol, patients complete PROMs every two months. However, post-trial, the frequency of measurements may vary between renal programs. In AKC-S, PROMs are administered electronically through a tablet and automatically entered into the local EMR. Electronic collection includes many considerations such as tablet purchase, configuration, storage, disinfection, and staff and patient comfort. Tablet displays had to be intuitive for clinicians and patients, and investments in ongoing information technology (IT) support and training were required. In AKC-N, patients complete PROMs on paper and nurses manually enter patients’ responses into the EMR. The collection process varied across units. Some units applied a schedule for PROMs administration, some coordinated PROMs to accompany blood draws, while some units employed a practice that patients’ primary nurse would administer and follow-up on PROMs. Allowing for variability within the local unit workflow was necessary for successful implementation.

### PROMs reporting

**Fig. 1:**
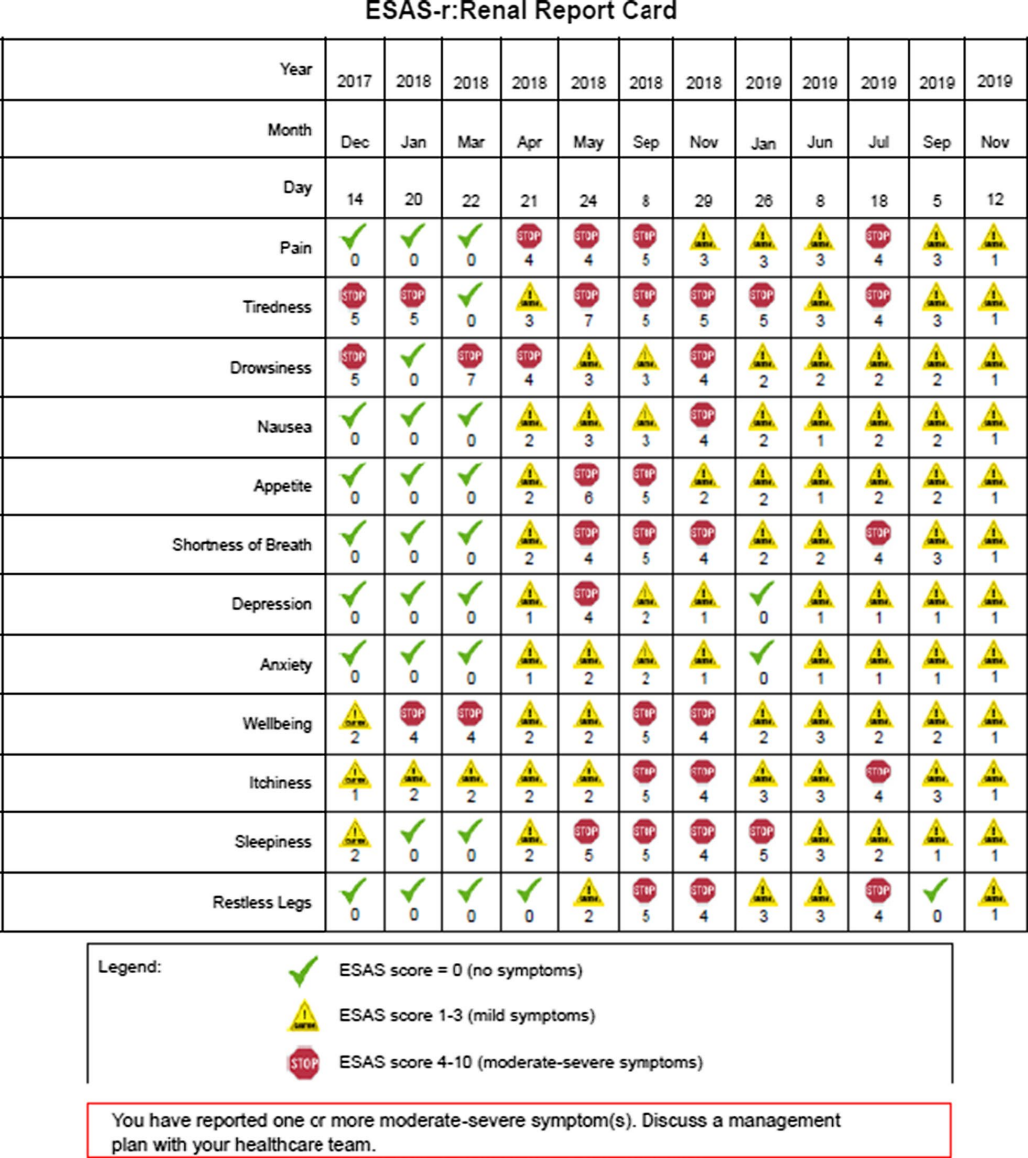
Sample ESAS-r: Renal PROMs report card

The implementation and integration of PROMs assessments into hemodialysis clinics requires a feasible system to capture and report PROMs. This was facilitated by the development of PROM report cards to present easily interpretable results to clinicians and patients, similar to clinical laboratory measures. Both renal programs’ EMRs generate a PROM report card with a symbol scheme for follow up (see Fig. 1 for sample). The symbol scheme comprised a check mark for no problems/symptoms (ESAS-r: Renal = 0, IPOS-Renal = 0, EQ-5D-5L = 1), a caution sign for mild problems/symptoms (ESAS-r: Renal = 1–3, IPOS-Renal = 1, EQ-5D-5L = 2), and a stop sign for moderate-severe symptoms/problems (ESAS-r: Renal = 4–10, IPOS-Renal = 2–4, EQ-5D-5L = 3–5). This symbol scheme is intended to help patients and nurses interpret PROMs scores and facilitate discussion for symptom management. The PROM report card is printed and added to the patient’s medical chart for review by clinicians (i.e., nurses, nephrologists, and allied health professionals). The report displays each patient’s most recent PROM scores in comparison with their previous scores. Patients are also offered a copy of their PROM report card.

### PROMs linkage to treatment aids

After PROMs are collected and report cards are reviewed and discussed with patients, clinicians can utilize treatment aids to manage symptoms. Treatment aids are actionable assessment and treatment resources developed by expert clinicians and patient partners for the management of certain symptoms. Several of the treatment aids were developed as part of the original supportive care program in AKC-N [7], but were modified, or new aids developed within each of the renal programs specific to symptoms assessed by the ESAS-r: Renal or IPOS-Renal (e.g., pruritis, restless legs, tiredness, nausea) and the EQ-5D-5L (e.g., pain, anxiety/depression)[24, 25]. Local guidance also reflected differences in the availability of resources (e.g., dieticians, social workers and spiritual care) and local patterns of practice (e.g., referrals and role of primary care). Patient-facing materials (i.e., patient handouts) were developed for patients to help them better understand the reasons why their symptom may have developed, treatment options, as well as self-care activities they can undertake to alleviate the symptom. Patient involvement in developing these materials was crucial for relevancy.

### Clinician training

**Fig. 2:**
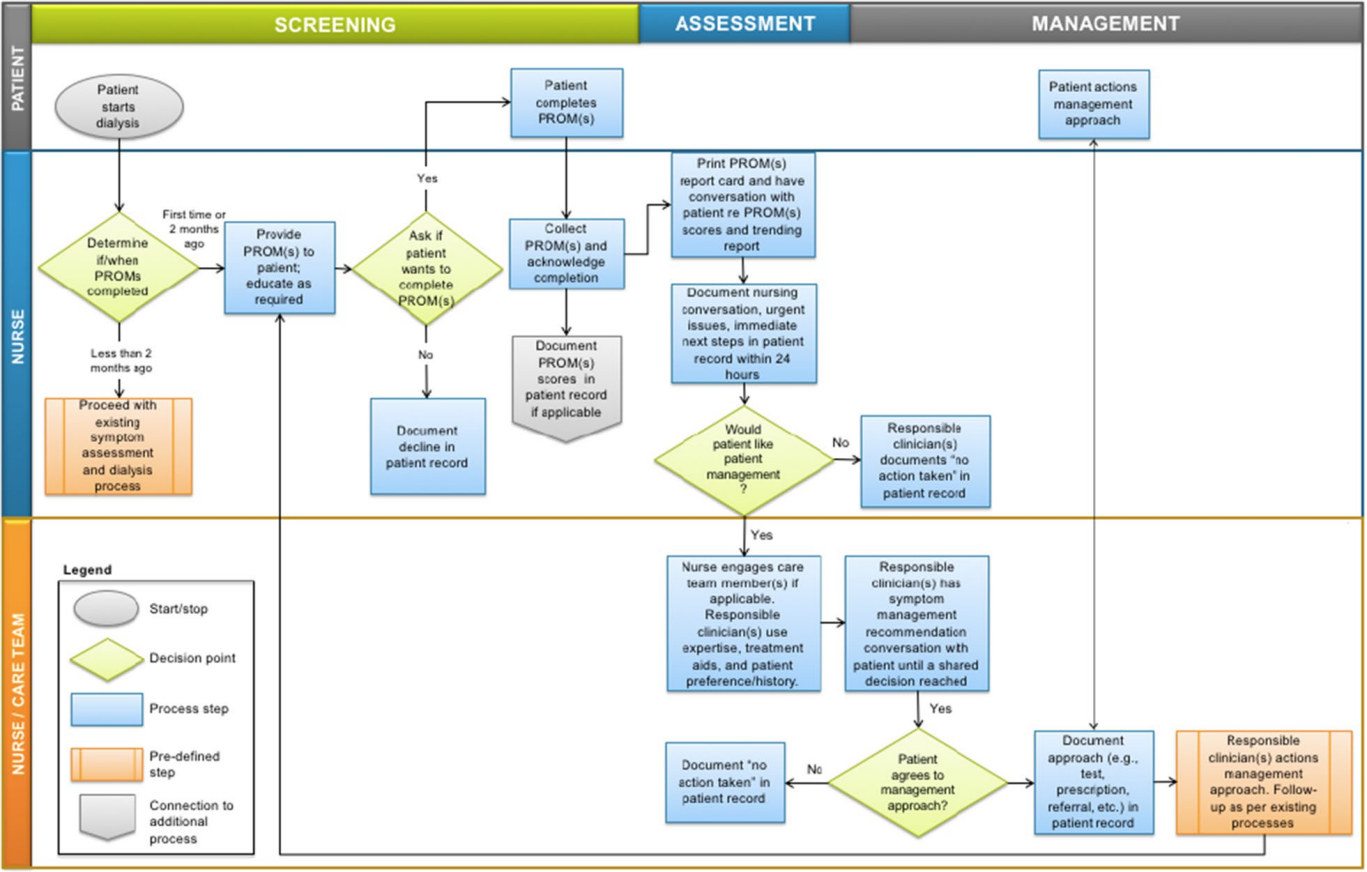
Implementation of PROMs into nurse workflow

Across AKC-N and AKC-S, nurses are largely responsible for the collection and follow-up of PROMs. They received training on the use of the ESAS-r: Renal or IPOS-Renal and the EQ-5D-5L including an overview of the instruments, interpretation of patients’ scores, changes in scores over time, use of PROMs data during clinical visits (i.e., referring to treatment aids), and EMR data entry. A toolkit for using PROMs in the hemodialysis unit was developed and shared with all clinicians in the hemodialysis units. A train-the-trainer model was adopted, where clinical nurse educators received training and subsequently provided on-site training to other nurses. Initial training was administered in a series of webinars and workshops over the course of two months. Figure 2 demonstrates the training workflow. Nephrologists also received training through in-person presentations and print materials. Additionally, each unit selected a nurse ‘site lead’ to champion the PROMs interventions and provide real-time feedback on the implementation to program managers. Ongoing training on using the treatment aids to manage specific symptoms was also provided as required to address specific concerns, or for staff orientation.

## Summary

The implementation of PROMs measurement and associated interventions can be challenging given the nature of clinical practice in busy hemodialysis units, the variations in organization and clinical workflow across units, as well as regional programs. Incorporating PROMs into clinical practice is an appropriate strategy to engage patients and enhance their role in decisions regarding their care and outcomes. However, it is not enough to simply collect PROM measurements from patients. Implementing PROMs and linking these with actionable treatment aids to alleviate bothersome symptoms and improve patients’ wellbeing is key to improving patients’ health. Other considerations in implementing PROMs within a hemodialysis setting include integration into electronic medical records, purchase and configuration of electronic tools (i.e., tablets), storage and disinfection of such tools, and ongoing IT resources. It is important to train clinicians on the practical elements of using PROMs, however there is also a need to engage clinicians to use PROMs on an ongoing basis.

The EMPATHY trial is intended to comprehensively evaluate the effectiveness of disease-specific and generic PROMs-based interventions, the adoption and implementation within real-world hemodialysis settings, as well as the cost-effectiveness of PROMs in routine hemodialysis care. Along with planned qualitative assessments, the information gained from this implementation trial will provide valuable information for the regional dialysis programs in enhancing and sustaining PROM interventions.

## Data Availability

Material are available from the corresponding author upon reasonable request and with permission of the University of Alberta.

## Abbreviations

AHS: Alberta health services
AKC-N: Alberta kidney care-north
AKC-S: Alberta kidney care-south
CKD: Chronic kidney disease
EMR: Electronic medical record
ESAS: Edmonton symptom assessment system
ESAS-r: RenalEdmonton symptom assessment system- revised: renal
HRQL: Health-related quality of life
IPOS: Integrated palliative care outcome scale
IPOS-Renal: Integrated palliative care outcome scale- renal
IT: Information technology
mESAS: Modified edmonton symptom assessment system
POS: Palliative care outcome scale
PROMs: Patient-reported outcome measures
