# Correction to: Patient-reported outcome measures in the care of in-centre hemodialysis patients

**DOI:** 10.1186/s41687-021-00388-w

**Published:** 2021-11-03

**Authors:** Sara N. Davison, Scott Klarenbach, Braden Manns, Kara Schick-Makaroff, Robert Buzinski, Bonnie Corradetti, Hilary Short, Jefrey A. Johnson

**Affiliations:** 1 Division of Nephrology and Immunology, Department of Medicine, University of Alberta, 11‑113L Clinical Sciences Building, Edmonton, AB T6G 2G3 Canada; 2 Department of Medicine and Department of Community Health Sciences, University of Calgary, O’Brien Institute of Public Health, 3280 Hospital Drive NW, Calgary, AB T2N 4Z6 Canada; 3 Faculty of Nursing, University of Alberta, 5‑295 Edmonton Clinic Health Academy, Edmonton, AB T6G 1C9 Canada; 4 Patient Partner, Medicine Hat, Alberta, Canada; 5 Patient Partner, Calgary, AB Canada; 6 Alberta PROMs and EQ‑5D Research and Support Unit, School of Public Health, University of Alberta, Shing Centre for Health Research Innovation, 2-040 Li Ka Shing Centre for Health Research Innovation, Edmonton, Alberta Canada

## Correction to: J Patient Rep Outcomes 2021, 5(Suppl 2):93 https://doi.org/10.1186/s41687-021-00365-3

After publication of this supplement [1], it was noticed that Dr. Sara Davison should be marked as the corresponding author instead of Dr. Braden Manns. Also, one of the author’s Family Names was misspelled: it should be read "Kara Schick-Makaroff" instead of "Kara Schnick-Makaroff”.

The authors names are correctly presented in the author list of this correction.

## References

[1] Davison (2021). The use of PROMs in health systems—implementation stories from Alberta, Canada. J Patient Rep Outcomes.

